# Developed Lower-Positioned Transverse Ligament Restricts Eyelid Opening and Folding and Determines Japanese as Being With or Without Visible Superior Palpebral Crease

**Published:** 2013-07-05

**Authors:** Midori Ban, Kiyoshi Matsuo, Ryokuya Ban, Shunsuke Yuzuriha, Ai Kaneko

**Affiliations:** ^a^Department of Plastic and Reconstructive Surgery, Shinshu University School of Medicine, Matsumoto; ^b^Department of Plastic and Reconstructive Surgery, Hamamatsu University School of Medicine, Hamamatsu, Japan

## Abstract

**Introduction:** We have reported that a developed lower-positioned transverse ligament between the superior-medial orbital rim and the lateral orbital rim on the lateral horn in the lower orbital fat space antagonizes eyelid opening and folding in certain Japanese to produce narrow eye, no visible superior palpebral crease, and full eyelid. In this study, we confirmed relationship between development of the lower-positioned transverse ligament and presence of the superior palpebral crease. **Methods:** We evaluated whether (1) digital immobilization of eyebrow movement during eyelid opening and (2) a developed lower-positioned transverse ligament could classify Japanese subjects as being with or without visible superior palpebral crease. **Results:** Digital immobilization of eyebrow movement restricted eyelid opening in all subjects without visible superior palpebral crease but did not restrict in any subject with visible superior palpebral crease. Macroscopic and microscopic evidence revealed that the lower-positioned transverse ligament behind the lower orbital septum in subjects without visible superior palpebral crease was significantly more developed than that in subjects with visible superior palpebral crease. **Conclusions:** Since a developed lower-positioned transverse ligament antagonizes opening and folding of the anterior lamella of the upper eyelid in subjects without visible superior palpebral crease, these individuals open their eyelids by lifting the eyebrow with the anterior lamella and the lower-positioned transverse ligament owing to increased tonic contraction of the frontalis muscle, in addition to the retractile force of the levator aponeurotic expansions. In subjects with visible superior palpebral crease, the undeveloped lower-positioned transverse ligament does not antagonize opening and folding of the anterior lamella, and so they open their eyelids by folding the anterior lamella on the superior palpebral crease via the retractile force of the levator aponeurotic expansions.

According to anthropological studies, the Japanese are genetically divided into the Neolithic Jomon natives and the Bronze Yayoi migrants from the cold Asian continent.^1^^-^6 The Yayoi migrants were reported to possess eyelid structures for cold tolerance, such as narrow eye, no visible superior palpebral crease (SPC), full eyelid, and high-positioned round supraorbital margin (Fig 1a), whereas the Jomon natives were reported to have features of wide eye, visible SPC, and low-positioned straight supraorbital margin (Fig 1b).

We have demonstrated that the presence of a developed lower-positioned transverse ligament (LTL) between the superior-medial orbital rim and the lateral orbital rim on the lateral horn in the lower orbital fat space, around which the superficial levator aponeurosis turns upwards to become the orbital septum, produces distinct features that include narrow eye, no visible SPC, and full eyelid, all of which correspond to features of the Yayoi migrants (Fig 1a).^7^^,^^8^ We hypothesized that a developed LTL in the lower fat pad space antagonizes opening of the eyelid, which leads to narrow eye, as well as folding of the anterior lamella of the upper eyelid owing to retraction of the levator aponeurosis expansions, which results in no visible SPC, and keeps the orbital fat in the lower position, which results in full eyelid (Fig 1a).^7^^,^^8^ To maintain an adequate visual field in primary gaze under these circumstances, increased contraction of the levator nonskeletal fast-twitch muscle fibers stretches the mechanoreceptors in Müller's muscle to enhance the levator skeletal slow-twitch muscle fibers and induce not only phasic, but also tonic, reflex contraction of the frontalis skeletal slow-twitch muscle fibers (Fig 1a).^8^^-^24 The tonic reflex contraction of the frontalis muscle persistently lifts the eyebrow,^23^ which carries both the anterior lamella of the upper eyelid and the LTL, even in primary gaze, and functions as another eyelid-opening mechanism (Fig 1a).

On the contrary, the absence of a developed LTL in the lower orbital fat space appears to produce certain features that include wide eye, visible SPC, and no full eyelid, all of which correspond to features of the Jomon natives (Fig 1b).^7^^,^^8^ We also hypothesized that an undeveloped LTL does not antagonize either opening of the eyelid, thus producing wide eye, or folding of the anterior lamella via retraction of the levator aponeurosis expansions, thereby resulting in visible SPC, and that movement of the orbital fat in the lower position precludes full eyelid (Fig 1b).^7^^,^^8^ To maintain an adequate visual field, Jomon natives need not persistently lift the eyebrow with the anterior lamella and weak LTL in primary gaze (Fig 1b).

Modern Japanese people continue to exhibit features of the Jomon natives or Yayoi migrants. Since the absence of visible SPC indicates the anterior lamella of the upper eyelid to be unfoldable and require lifting of the eyebrow for maintenance of an adequate visual field, we considered this to be the key distinguishing trait of descendants of the Yayoi migrants in this study. In contrast, we presumed the presence of visible natural SPC to specifically indicate the anterior lamella of the upper eyelid as being foldable on the SPC without lifting the eyebrow for maintenance of an adequate visual field in Japanese stemming from Jomon native ancestry.

Digital immobilization of eyebrow movement during eyelid opening from closed eyelid to primary gaze counteracts the contraction of the frontalis muscle to lift the eyebrow with the anterior lamella of the upper eyelid and is commonly used to exclude involvement of eyebrow lifting when impaired levator function is evaluated in congenital blepharoptosis (Fig 2c). This technique was also used to observe how the LTL antagonizes eyelid opening in our cohort.

To verify our hypotheses, we evaluated whether (1) digital immobilization of eyebrow movement during eyelid opening and (2) a developed LTL that restricts eyelid opening and folding could classify Japanese subjects as being with or without visible SPC.

## SUBJECTS AND METHODS

We enrolled 66 Japanese subjects (53 women and 13 men, aged 18-79 years). Reasons for the surgery consisted of 10 blepharoplasties, 18 bilateral acquired blepharoptoses, and 5 unilateral congenital blepharoptoses in 33 subjects without visible SPC, and 8 blepharoplasties and 25 bilateral acquired blepharoptoses in 33 subjects with visible natural SPC. The nonptotic eyelids in 5 subjects with unilateral blepharoptosis were evaluated.

We first evaluated whether digital immobilization of eyebrow movement by pressing on the anterior surface of the supraorbital margin, during movement from loosely closed eyelids following tight eyelid closure with relaxing the frontalis muscle^23^ to eyelid opening for primary gaze, restricted eyelid opening in both groups (Figs 2c, 3b, 4b, and 5b). A pupillary center that was not exposed under digital immobilization was judged to be restricted, while one that was exposed was judged as unrestricted.

Intraoperatively, LTLs in the lower orbital fat space were analyzed in terms of size and variation based on an attached 10-mm^2^ square scale (Casmatch; Dai Nippon Printing Co, Ltd, Tokyo, Japan) and the size of retractors or forceps (Figs 2d, 2e, 3c, 4c, 5c, and 6). The width of the lowest LTL in each subject was measured for statistical comparisons of both group subjects without and with visible SPC (Fig 7). Three representative LTLs in each group that were resected for surgical purposes were subjected to Azan staining for histological analysis (Fig 8).

All subjects gave informed consent to participate in this study, which was approved by our institutional review board for human subjects. Statistical analysis was performed using the Student *t* test. *P* < .05 was used to indicate statistical significance.

## RESULTS

Digital immobilization of eyebrow movement restricted eyelid opening in all 33 subjects without visible SPC (Figs 2c and 3b) but did not restrict in any subject with visible SPC, even in those with acquired blepharoptosis (Figs 4b and 5b).

Lower-positioned transverse ligaments behind the lower orbital septum consisted of 1 thick LTL in 23 (Fig 3c) and 2 or more thick LTLs in 10 (Fig 2e) of the 33 subjects without visible SPC. Lower-positioned transverse ligaments consisted of 1 thin LTL in 18 (Figs 4c and 5c) and 2 or more thin LTLs in 13 of the 33 subjects with visible SPC (Fig 6) and were undetectable in 2 subjects in this group.

The mean width of the lowest LTL behind the lower orbital septum in the nonvisible SPC group (1.15 ± 0.44 mm) was significantly larger than that in the visible SPC group (0.88 ± 0.45 mm) (*P* = 0.0136) (Fig 7).

Histological examination confirmed our macroscopic findings, whereby collagen fibers of the LTL in subjects without visible SPC (Fig 8a) appeared to be thicker than those in subjects with visible SPC (Fig 8b).

## DISCUSSION

**Figure 1:**
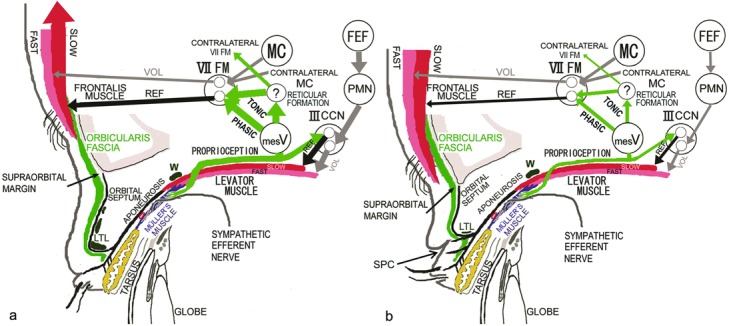
Anatomy and neurophysiology related to eyelid opening. (*a*) Subjects without visible SPC or Jomon natives. (*b*) Subjects with visible SPC or Yayoi migrants. ? indicates unknown nucleus; FAST, fast-twitch muscle fibers; FEF, frontal eye field; III CCN, central caudal nucleus of the oculomotor nuclear complex; LTL, lower-positioned transverse ligament; mesV, mesencephalic trigeminal nucleus; PHASIC, phasic contraction; PMN, premotor neurons in the rostral interstitial nucleus of the medial longitudinal fasciculus; REF, reflex contraction; SLOW, slow-twitch muscle fibers; SPC, superior palpebral crease; TONIC, tonic contraction; VII FM, frontalis motor neurons; VOL, voluntary contraction; W, Whitnall's ligament.

**Figure 2:**
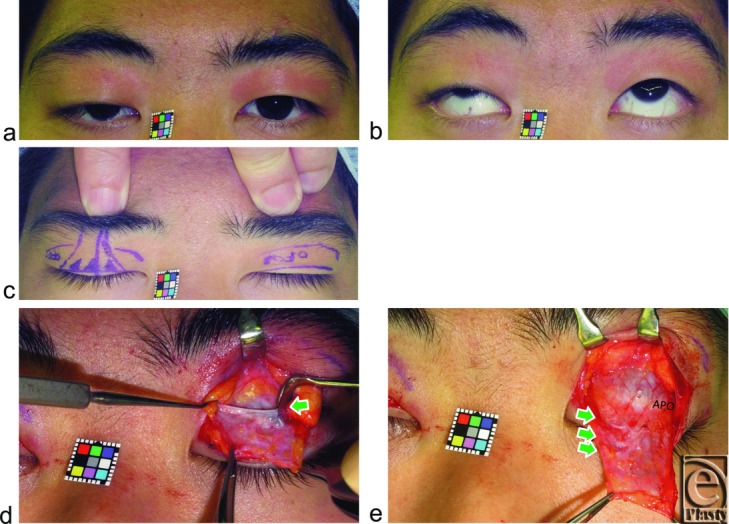
A 23-year-old man without visible SPC suffering from right congenital blepharoptosis. (*a*, *b*) Primary and upward gaze. (*c*) Digital immobilization of eyebrow movement restricts opening of not only the congenitally ptotic right eyelid but also the normal left eyelid. (*d*) Arrow indicates the lowest LTL between the superior-medial orbital rim and the lateral orbital rim on the lateral horn in the lower orbital fat space. (*e*) Arrows indicate 3 LTLs. Abbreviations are explained in the caption of Figure 1.

**Figure 3:**
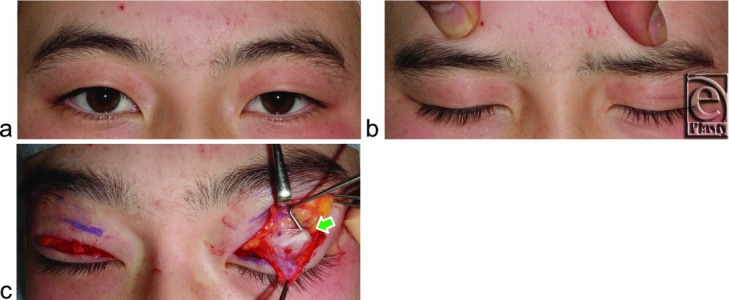
An 18-year-old woman without visible SPC being treated for blepharoplasty. (*a*) Primary gaze. (*b*) Digital immobilization of eyebrow movement completely restricts opening and folding of both eyelids. (*c*) Arrow indicates 1 thick LTL behind the orbital septum. Abbreviations are explained in the caption of Figure 1.

**Figure 4:**
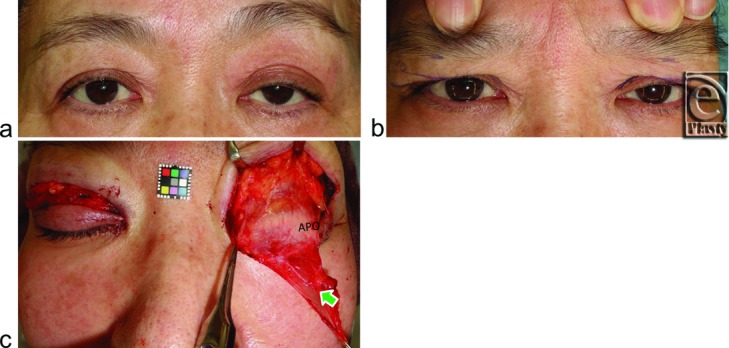
A 59-year-old woman with visible SPC suffering from left acquired blepharoptosis owing to the use of unilateral contact lens for 40 years. (*a*) Primary gaze. (*b*) Digital immobilization of eyebrow movement does not restrict opening and folding of either eyelid. (*c*) Arrow indicates a thin LTL. APO indicates aponeurosis. Other abbreviations are explained in the caption of Figure 1.

Macro- and microscopic evidence obtained in the current study demonstrated that whereas subjects without visible SPC had a developed LTL behind the lower orbital septum in terms of not only the width of the lowest LTL and the number of LTLs (Fig 1a), those with visible SPC had an undeveloped LTL (Fig 1b). Furthermore, digital immobilization of eyebrow movement revealed that the developed LTL strongly antagonized both opening and folding of the anterior lamella, which was not observed in undeveloped LTL subjects with visible SPC.

**Figure 5:**
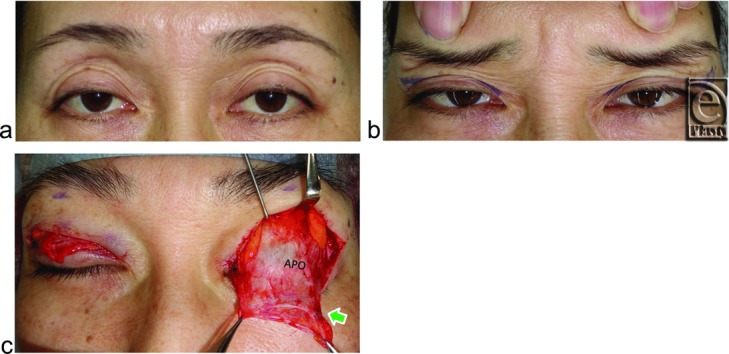
A 45-year-old woman with visible SPC suffering from bilateral acquired blepharoptosis owing to the use of bilateral contact lens for 27 years. (*a*) Bilateral eyebrow are lifted by compensatory reflex contraction of the frontalis muscles in primary gaze. (*b*) Digital immobilization of the eyebrow movement slightly restricts opening of eyelids but does not restrict folding of eyelids. (*c*) An arrow indicates a thin LTL. APO indicates aponeurosis. Other abbreviations are explained in the caption of Figure 1.

**Figure 6:**
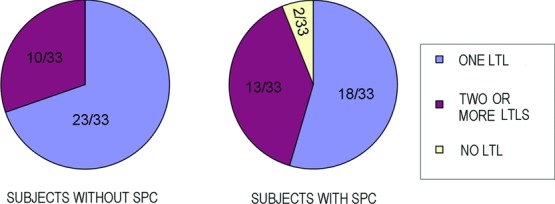
Variations in numbers of LTLs in subjects without or with SPC. Abbreviations are explained in the caption of Figure 1.

**Figure 7:**
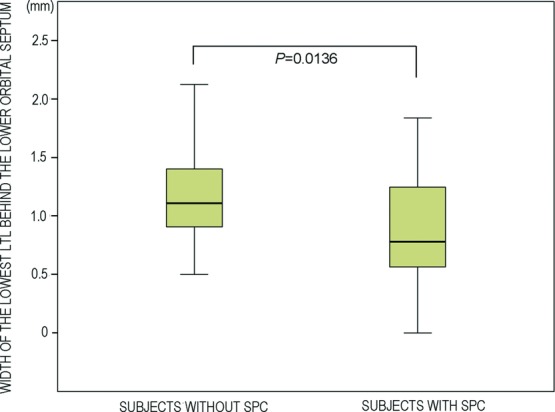
Difference in width of the lowest LTL between subjects without and with visible SPC. Abbreviations are explained in the caption of Figure 1.

**Figure 8:**
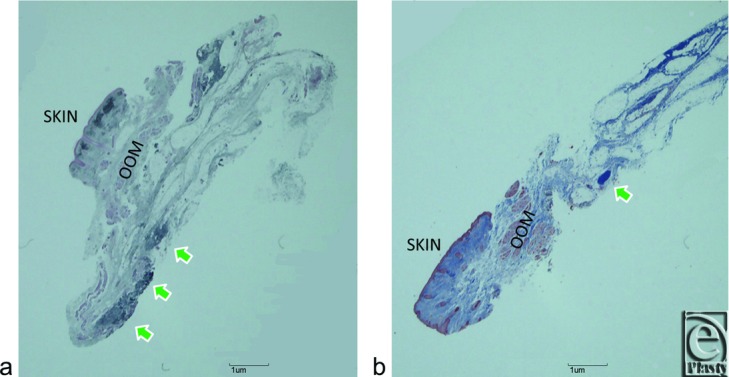
Azan-stained sagittal sections of surgically discarded tissues that include LTL in representative subjects without (*a*) or with (*b*) SPC. Green arrows indicate LTLs. OOM indicates orbicularis oculi muscle. Other abbreviations are explained in the caption of Figure 1.

On the basis of our findings, it appears that variations in the LTL may determine the features of Yayoi migrants or the Jomon natives in the Japanese. A developed LTL between the superior-medial orbital rim and the lateral orbital rim on the lateral horn behind the lower orbital septum not only restricted eyelid opening and folding but also kept the orbital fat in a lower position. Subsequently, narrow eye, no visible SPC, and fullness of the upper eyelid ensued as specific features of the Yayoi migrants. They appeared to open the eyelid by not only the eyelid retraction but also the upward movement of the lateral canthus. On the contrary, an undeveloped LTL did not restrict eyelid opening and folding, resulting in the wide eye and visible SPC that are distinctive of the Jomon natives. Undeveloped LTL might allow the orbital fat to sink into the upper orbit like the Occidental eyelid (Fig 5). In the Occidental eyelid, a “white line” has been reported in some patients with acquired blepharoptosis that was located where we identified the LTL in our subjects with visible SPC.^25^ However, it has also been suggested that this line represents the rolled and retracted fibers of the stretched levator aponeurosis.

In subjects without visible SPC, because the developed LTL antagonizes opening and folding of the anterior lamella of the upper eyelid, these people open their eyelids not only by the retractile force of the levator aponeurotic expansions but also by lifting the eyebrow with the anterior lamella and developed LTL owing to increased tonic contraction of the frontalis muscle. Since their eyebrows had been lifted in normal primary gaze, the digital immobilization test in this study was performed during movement from loosely closed eyelids following tight eyelid closure with relaxing the frontalis muscle to eyelid opening for primary gaze. In subjects with visible SPC, because the undeveloped LTL did not antagonize opening and folding of the anterior lamella, they were able to open their eyelids by folding the anterior lamella on the SPC via the simple retractile force of the levator aponeurotic expansions.

Our subjects who had no visible SPC and developed LTL may correspond to the Yayoi migrants with eyelid structures for cold tolerance. Because they always lift the eyebrows in primary gaze, their supraorbital margin may be high-positioned and round as a result of the lifting force by tonic contraction of the frontalis muscle, which mechanically presses on the supraorbital margin (Fig 1a). In contrast, the subjects who had visible SPC and undeveloped LTL may correspond to the Jomon natives without eyelid structures for cold tolerance. As they do not lift the eyebrows on primary gaze, their supraorbital margin is presumed to be low positioned and straight (Fig 1b).

## CONCLUSIONS

The development of LTL restricts eyelid opening and folding and distinguishes Japanese as being with or without visible SPC, or rather being of Jomon native or Yayoi migrant ancestry. To compensate for the restriction of eyelid opening and folding in Japanese without SPC, tonic reflex contraction of the frontalis muscle to persistently lift the eyebrow with the anterior lamella of the upper eyelid and thick LTL serves as another eyelid-opening mechanism. From a surgical viewpoint, both the excision of LTLs behind the orbital septum and the creation of the functional SPC, on which the anterior lamella is folded, may reduce both tonic reflex contraction of the frontalis muscle and the eyebrow height. Further studies are needed on the differences in supraorbital margin shape between Japanese subjects without and with visible SPC, as well as on the relationship between LTL development and expansion of the levator aponeurosis to the pretarsal skin, both of which contribute to the formation of SPC.
